# Digital Interventions on Healthy Lifestyle Management: Systematic Review

**DOI:** 10.2196/26931

**Published:** 2021-11-17

**Authors:** Ayan Chatterjee, Andreas Prinz, Martin Gerdes, Santiago Martinez

**Affiliations:** 1 Department for Information and Communication Technologies Centre for e-Health University of Agder Grimstad Norway; 2 Department of Health and Nursing Science Centre for e-Health University of Agder Grimstad Norway

**Keywords:** eHealth, digital intervention, lifestyle, obesity, challenges, mobile phone

## Abstract

**Background:**

Digital interventions have tremendous potential to improve well-being and health care conveyance by improving adequacy, proficiency, availability, and personalization. They have gained acknowledgment in interventions for the management of a healthy lifestyle. Therefore, we are reviewing existing conceptual frameworks, digital intervention approaches, and associated methods to identify the impact of digital intervention on adopting a healthier lifestyle.

**Objective:**

This study aims to evaluate the impact of digital interventions on weight management in maintaining a healthy lifestyle (eg, regular physical activity, healthy habits, and proper dietary patterns).

**Methods:**

We conducted a systematic literature review to search the scientific databases (Nature, SpringerLink, Elsevier, IEEE Xplore, and PubMed) that included digital interventions on healthy lifestyle, focusing on preventing obesity and being overweight as a prime objective. Peer-reviewed articles published between 2015 and 2020 were included. We used the PRISMA (Preferred Reporting Items for Systematic Reviews and Meta-Analyses) guidelines and a framework for an evidence-based systematic review. Furthermore, we improved the review process by adopting the Rayyan tool and the Scale for the Assessment of Narrative Review Articles.

**Results:**

Our initial searches identified 780 potential studies through electronic and manual searches; however, 107 articles in the final stage were cited following the specified inclusion and exclusion criteria. The identified methods for a successful digital intervention to promote a healthy lifestyle are self-monitoring, self-motivation, goal setting, personalized feedback, participant engagement, psychological empowerment, persuasion, digital literacy, efficacy, and credibility. In this study, we identified existing conceptual frameworks for digital interventions, different approaches to provide digital interventions, associated methods, and execution challenges and their impact on the promotion of healthy lifestyle management.

**Conclusions:**

This systematic literature review selected intervention principles (rules), theories, design features, ways to determine efficient interventions, and weaknesses in healthy lifestyle management from established digital intervention approaches. The results help us understand how digital interventions influence lifestyle management and overcome the existing shortcomings. It serves as a basis for further research with a focus on designing, developing, testing, and evaluating the generation of personalized lifestyle recommendations as a part of digital health interventions.

## Introduction

### Overview

Deaths caused by lifestyle diseases are increasing rapidly compared with deaths caused by infectious disease [1-4]. Most lifestyle diseases arise from unhealthy and sedentary lifestyles, low nutritional propensities, and poor living conditions, affecting individuals from different financial backgrounds, beyond age and gender biases [1-3]. Lifestyle diseases are a monetary burden to individuals, families, businesses, and governments. They cause 41 million deaths each year, equivalent to 71% of all deaths worldwide [1-6]. Every year, 15 million people die from lifestyle diseases between the ages of 30 and 69 years, and more than 85% of these *premature* deaths occur in low- and middle-income countries [1-6]. The fundamental risk factors [7-13] behind lifestyle diseases are excessive alcohol and tobacco consumption, improper food plan, and physical inactivity, resulting in excess weight gain (obesity), increased blood glucose, hypertension, high blood cholesterol, and social detachment [7-13]. Obesity is a primary lifestyle disease that leads to other lifestyle diseases, such as cardiovascular diseases (CVDs), clinical obstructive pulmonary disease, cancer, type 2 diabetes, hypertension, and depression [7-13]. In 2016, more than 1.9 billion adults age ≥18 years were overweight. Of these, over 650 million were obese [2-4]. In 2016, 39% of adults aged ≥18 years were overweight, and 13% were obese [2-4]. In 2019, an estimated 38.2 million children aged ≤5 years were overweight or obese [2-4]. Once considered a problem in high-income countries, overweight and obesity are now rising in low- and middle-income countries (especially in urban environments) [2-4]. Since 2000, the number of overweight children aged ≤5 years has increased by nearly 24% in Africa [2-4,8]. In 2019, almost half of the children aged ≤5 years who were overweight or obese lived in Asia [2-4,8]. Thus, control plans need to include detection, screening, treatment, and prevention methods. Digital interventions may provide viable and hypothetically cost-effective models to improve well-being. They provide widely distributed, trusted, and personalized well-being information and services to fulfill individualized needs to maintain a healthy lifestyle [14-19].

Digital interventions for changing negative health behaviors to advance a healthy lifestyle are instigated by persuasion studies. The World Health Organization (WHO) has classified digital health interventions into the following 4 categories: clients, health care providers, health system managers, and data services, where digital and mobile technologies are being used to help well-being system needs and achieve health objectives [20-26]. Digital intervention methods include conceptualization, intervention strategies, policy design, understanding of the environment, motivation, behavioral determinants and psychology, persuasion, self-determination theory, self-regulation, participation (engagement), decision-making and feedback generation, goal setting and evaluation, incorporation of digital technologies (eg, smartphones, computers, and wearable sensors), and digital recommendation generation. Its success depends on credibility, satisfaction, privacy, digital literacy, proper connectivity, cocreation, and efficacy evaluation [20-26]. According to the WHO, harnessing the power of digital technology is critical to achieving universal health coverage, and digital technologies are not an end in themselves; they are essential tools for promoting health, maintaining world security, and serving the disadvantaged [18-24]. Digital interventions have been carried out effectively for well-being advancement and psychological well-being and for enabling self-administration of enduring conditions [18-24]. Nonetheless, their capability is restricted by low use rates, with noncommitment being a significant challenge. The use of digital technology provides new opportunities to improve health [18-24]. However, the evidence also highlights the challenges posed by certain interventions. If digital technologies are to be maintained and integrated into health systems, they must be able to demonstrate long-term improvements compared with traditional methods of providing health services [23,24].

### Limitations

Digital intervention depends heavily on the context and ensures the proper design, including the structural issues in the environment in which they are used, the available infrastructure, the health needs they are addressing, and the ease of use of technology [1,15-17]. Consequently, it is important to discover successful procedures for expanding individual engagement with digital interventions. Digital interventions can also support health workers to give them more opportunities to clinical protocols around, for example, decision support mechanisms or telemedicine consultation [15-17]. Digital interventions should supplement and enhance the functions of the health system by accelerating information exchange and other mechanisms but cannot substitute the essential components required by the health system, such as the health workforce, funding, leadership and governance, and access to critical medicines. However, digital interventions in health care have tremendous potential as scalable tools to enhance well-being and health care service conveyance by improving viability, proficiency, openness, and personalization [1,15-17]. The WHO is working hard to ensure that it can be used as efficiently as possible, which means adding value to medical staff and individuals who use these technologies, considering the limitations of the infrastructure, and making appropriate coordination [22-24]. Although digital intervention's application area is broad, we have focused only on *digital behavioral intervention for healthy lifestyle* studies in this paper.

### Study Aim

Efficiency, acceptability, and compliance are 3 necessary indicators of digital behavioral interventions, as they are prerequisites for positively affecting health or healthy behavior. Efficacy refers to the effect of using technology in behavioral interventions. Acceptability means that users are satisfied with the technology. Compliance refers to the degree to which technology is used, as expected. This systematic literature review addresses the following research questions (RQs):

RQ1: what are the existing conceptual frameworks for digital interventions for healthy lifestyle management?

RQ2: what are the different *approaches* to provide digital interventions for healthy lifestyle and what are the essential *methods*?

RQ3: what is the importance of digital intervention in promoting healthy lifestyles targeting obesity and overweight?

## Methods

### Overview

We used a systematic literature review method to obtain a broad overview of the current literature on the subject in a reproducible and understandable manner. A systematic review is a study of the evidence of a clearly expressed problem. It uses systematic and transparent strategies to understand, select, and strictly evaluate related basic research and extract and explore facts from the research covered in the evaluation. Systematic reviews represent scientific synthesis of evidence. We used the PRISMA [27] and Rayyan [28] evidence-based frameworks for systematic literature reviews. Subsequently, for article selection, we used the Scale for the Assessment of Narrative Review Articles (SANRA) scaling [29]. We conducted our systematic literature review following the Wendler [30] proposals to search scientific databases, as explained in *Strategy* subsection.

### Strategy

The system’s search strategy was designed using a combination of thesaurus, Oxford dictionary, and free terms covering the following terms: *eCoach, e-Coach, eHealth*, *e-Health*, *electronic* *coaching*, *online*, *automatic*, *persuasion*, *persuasive technology*, *mHealth*, *mobile* *health*, *digital health*, *mobile*, *digital* *intervention*, *smartphone*, *smart*-*phone*, *application*, *app*, *prevention*, *healthy* *behaviour*, *healthy* *behavior*, *behaviour* *change*, *behavior* *change*, *exercise*, *activity, walk, step, fitness, sitting, inactive, screen time, sport, leisure activity, nutrition, nutritional, diet, dietary, healthy eating, salad, vegetables, fruit, discretionary food, snack, sweet beverage, carbonated beverage, soft drink, habit, tobacco, alcohol, computer, sedentary, lifestyle, intervention, lifestyle recommendation, digital recommendation, behavioral recommendation, program, programme, conceptual model, health promotion, prevention, obesity, overweight, weight-gain, weight gain, weight change, engagement, effectiveness, efficiency, credibility, trust, motivation, regulation, challenges, preferences, sample,* and *study duration*. We filtered our search with the following search string: ((*eCoach* OR *Coach OR *coaching OR *automatic* OR *online* OR *eHealth* OR *e-Health* OR *persuasion* OR persuasive* OR *mHealth* OR mobile* OR *digital* *health*) AND (*digital* *intervention* OR *smartphone* OR *smart-phone* OR *computer* OR app* OR *prevention* OR *recommendation) AND (*behavior* OR *behaviour* OR sedentary* OR *lifestyle* OR *exercise* OR *activity OR *walk* OR *step* OR *fitness* OR *inactive* OR *screen* *time* OR *sport* OR nutrition* OR diet* OR *healthy* *eating* OR *salad* OR veg* OR *fruit* OR discretionary* OR *snack* OR *beverage OR *soft* *drink* OR *habit* OR *tobacco* OR *alcohol*) AND (*engagement* OR *persuasion* OR *effectiveness* OR *efficacy* OR *efficiency* OR *motivation OR *regulation OR challenge* OR limitation* OR *credibility* OR *trust* OR *preferences* OR *program* OR *programme* OR conceptual* OR *sample* OR *duration) AND (*obesity* OR *weight*)). We conducted the search in collaboration with the library of the University of Agder in Norway on the following 5 electronic databases including Nature, SpringerLink, Elsevier, IEEE Xplore, and PubMed, as they produced the maximum number of scientific sources related to digital intervention studies for healthy behavior targeting on the prevention of obesity and overweight as a primary objective. Related search keywords were identified using Medical Subject Headings terms, keywords from relevant articles, synonyms, and self-established search terms. *EndNote (V.9.x)*, *DOAJ*, *SHERPA/RoMEO*, and *Microsoft Excel (Office 365, 2019)* were used to search, collect, and select related articles effectively.

Studies on digital intervention are promising and have an enormous scope in the health care domain with information and communication technologies. A systematic literature review produced related older studies, but they are primarily in the theoretical phase, and the practical implementation is very young. Therefore, to keep our systematic literature review focused, articles related to digital interventions for healthy lifestyle management were included when published between January 1, 2015, and December 15, 2020. The search was limited to English literature, humans, digital health intervention methods, and research focused on improving healthy lifestyles. We aim to include peer-reviewed articles that describe digital intervention methodologies, conceptual models, theories, key challenges, lifestyle recommendations, and research related to healthy lifestyle management focusing on preventing obesity and being overweight with digital means. Articles are classified into the following groups: quantitative, qualitative, both quantitative and qualitative, and short papers, such as posters, editorials, and commentaries. Quantitative analysis is the factual examination of information gathered by the framework to test explicit speculations. Qualitative analysis centers around words and implications to investigate thoughts and encounters inside and out. The selection criteria (or specific parameters) for the quantitative and qualitative articles were (1) articles associated with a healthy lifestyle, with the main goal of preventing obesity and being overweight using digital interventions (or recommendations); (2) methods, theories, and strategies associated with digital interventions; and (3) challenges of digital interventions for lifestyle change.

We aim to adopt the explicit inclusion and exclusion criteria, as described in Textbox 1 and divide and distribute the articles among authors to complete the screening using the *Rayyan* collaboration and research tool. After individual screening, the results will be verified by other authors to resolve discrepancies between the reviewers. Subsequently, eligible peer-reviewed articles will be identified by manual search, quality score, and manual assessment of reference lists of related papers. Initially, titles, keywords, abstracts, and conclusions will be screened for inclusion. Then, we review the screened articles independently and check for individual eligibility for final inclusion.

**Inclusion and exclusion criteria for systematic literature review.**

**Inclusion criteria**

- Peer-reviewed, full-length articles written in English
- Digital intervention on healthy lifestyle articles published in the selected databases between 2015 and 2020
- Articles indexed in *Google Scholar* and (*SCOPUS* or *SCI* or *SCIE*)
- Journal papers, conference papers, or books
- Both qualitative (primary and secondary research) and quantitative studies
- *Open access* and accessible through the university library
- Studies associated with behavior change prevention rather than treatment and management of health conditions

**Exclusion criteria**

- Articles not written in English
- Incomplete, non–peer-reviewed articles
- Nonexperimental studies
- Poster, editorial, and commentary papers
- Articles published outside the selected time frame (<2015 and >2020)
- Articles not indexed in *Google Scholar* and (*SCOPUS* or *SCI* or *SCIE*)
- Studies related to offline human behavior interventions
- Papers having a considerable amount of analogous content or exact duplicate articles
- Economic, investment, and policy-making articles related to digital interventions
- Articles related to impacts from social interactions on behavior changes
- Articles related to traditional nutritional and physical activity assessment without any adoption of digital intervention methodologies
- Articles related to behavioral impacts on other lifestyle diseases apart from obesity such as cancer, mental health, chronic obstructive pulmonary disease, cardiovascular disease, dysglycemia, type 2 diabetes, loneliness, and hypertension
- Articles related to the treatment and management of health conditions rather than prevention of lifestyle disease (obesity and overweight in context)
- Articles related to cultural adaptation, nutrition policy, pregnancy, and genetics
- Articles related to worksite wellness, remote patient monitoring, hospital care, osteoporosis prevention for older adults, and web-based intervention for victims of cyberbullying

**Figure 1:**
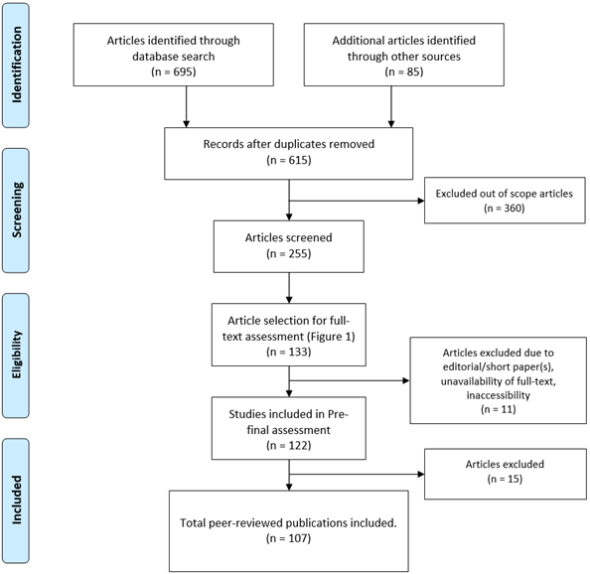
PRISMA (Preferred Reporting Items for Systematic Reviews and Meta-Analyses) flowchart for article selection process.

The search results from the databases for full-text assessment are as follows: IEEE Xplore (n=2), Nature (n=4), Elsevier (n=28), PubMed (n=40), and SpringerLink (n=59); “n” signifies total number articles screened for full-text assessment. We then excluded short research articles (1-2 pages) from the previous search list (Figure 1). In the prefinal assessment, for data extraction, we maintained an Excel spreadsheet with the following fields: title, reference in the American Medical Association format, author, population size, study duration, target group (children, adolescents, adults, and older adults), nature of the paper (review, conceptual or methodology, survey, and implementation), year, country of research, key terms, keywords, intervention type, publication channel, technology use, peer-reviewed, key findings (outcome, measures, intervention methods, theory, intervention components, effectiveness, and results), nature of assessment (qualitative, quantitative, or both), key challenges, and quality score based on SANRA. The primary outcome indicators extracted from the preliminary research results were digital intervention methods, nutritional intake, physical exercise, and healthy habits (consumption of tobacco and alcohol). The quality of the included articles was assessed using the SANRA 0-2-point scale. We graded individual papers based on the 6 quality parameters, as defined in Textbox 2. Individual quality parameters were subcategorized into a 0-2–point scale. Finally, we calculated the mean score of the 6 quality parameters. The number of studies included in the prefinal assessment was 122 with a SANRA score >1.90. Out of 122 studies, 15 had a SANRA score between 1.9 and 2.0. Therefore, we selected 107 articles that scored scale 2 based on the SANRA scale (Textbox 2).

**Searched article scaling based on quality parameters.**

**Quality parameters**

- Justification of the article’s importance for readership
- Statement of concrete or specific aims or formulation of questions
- Conference and journal papers to describe the overview of this study
- Referencing
- Scientific reasoning
- Appropriate presentation of data

In the final stage, we had articles with grade 2 and cited them as references (Textbox 3). In addition, we added 30 articles to the reference list (Textbox 3), including websites (accessed URLs), conference and journal papers to describe the overview of this study, risks of lifestyle diseases, and healthy behavior plans. The complete process of choosing the source for this study is shown in the flowchart in Figure 1. The article selection process consists of four stages—identification, screening, eligibility, and inclusion. Instead of depicting all the included studies as separate tables, we have presented significant findings from the respective studies.

**Nature of studies in the reference list.**

**Nature of studies**

- Articles and web resources (URLs) to describe the overview of this study, risks of lifestyle diseases, and healthy behavior plan [2-31]
- Included studies with the Scale for the Assessment of Narrative Review Articles scale 2 (107 articles) [1,32-137]

## Results

### Study Selection and Study Characteristics

The searches (electronic database and manual databases) resulted in 780 papers (695 in electronic databases and 85 manually), of which 165 were duplicates. A total of 360 articles were excluded from the study following the exclusion criteria, and 255 articles were screened by reading abstracts, keywords, and conclusion sections. We selected 133 articles for full-text reading. We decided on 122 articles after a full-text review and checked the paper's length and the full-text availability in the prefinal stage. The final search produced 107 core peer-reviewed articles eligible for citation (4 from Nature, 49 from Springer, 23 from Elsevier, 1 from IEEE, and 30 from PubMed) related to digital intervention for healthy lifestyle management. Of the 107 papers, 72 (67.3%) were qualitative, 29 (27.1%) were quantitative, and the remaining 6 were both qualitative and quantitative. The final 107 papers were selected from 4 continents, including Asia, Europe, North America, and Oceania with the subsequent detailed search results as indicated by “n”: Europe (n=56), North America (n=32), Oceania (n=16), Asia (n=2), Asia and North America (n=1), North America and Europe (n=1), and Oceania and North America (n=1).

The selected studies were clustered among the following 4 study groups that helped to answer our RQs: review (n=38), implementation (n=37), conceptual or methodology (n=23), and survey (n=9) studies related to digital interventions for healthy lifestyle management. Here, *n* signifies the total distribution of studies in the 4 study groups.

The findings related to the identified RQs are elaborated as follows.

### RQ1: What Are the Existing Conceptual Frameworks for Digital Interventions for Healthy Lifestyle Management?

Mummah et al [32] proposed the concept of a conceptual framework with the following 10 phases: empathize with target users, define the target behavior, the basics of behavioral theory, come up with implementation strategies, potential prototype products, gather user feedback, build a real minimum product, a pilot test to evaluate potential efficacy and utility, evaluation of effectiveness in randomized controlled trials, and sharing of interventions and results. These phases are grouped into 4 overarching categories: integration, design, evaluation, and sharing.

Muench et al [33] proposed an overarching framework to perform digital triggering (text messages, emails, and push alerts) focusing on individual goals with the following 5 components: who (sender), how (stimulus type, delivery medium, and heterogeneity), when (delivered), how much (frequency and intensity), and what (trigger target, trigger structure, and trigger narrative). They showed how user characteristics, conceptual models, and clinical aims help to plan digital interventions and initiate tailoring with product features and user states.

Lewis et al [34] provided an idea to understand human behavior technology engagement to measure digital behavior change interventions (DBCIs) using a proposed framework. The proposed framework conceptualizes the 2 basic categories of commitment measured in digital behavior interventions (DBIs). The types are committed to health behaviors known as *Big E* and involvement of DBI known as *Small E.* DBI engagement has been further broken down into 2 subclasses: user interactions with intervention features designed to encourage frequent use, such as simple log-in, games, and social interactions and make the user experience attractive, and interactions of the user with the components of a behavior change intervention (ie, behavior change techniques) that influence determinants of health behaviors and then affect health behaviors.

Wang et al [35] proposed a holistic TUDER (Targeting, Understanding, Designing, Evaluating, and Refining) framework to integrate taxonomies into the theory-based digital health behavior intervention model. They showed how digital health behavior intervention is guided and influenced by theoretical concepts, such as behavior theories, behavior change technologies, and persuasive technology.

Lubans et al [36] proposed a framework for designing and delivering organized physical activity (PA) sessions for children and adolescents for effective dissemination. Recommended strategies include creating partnerships, presentations, intervention dissemination, scaling up research, and embedding evidence-based interventions.

According to Morgan et al [37], the limitations of digital health intervention programs include the lack of attention to critical sociocultural factors that affect participation and interventions on research results. Their research provides a conceptual model that illustrates the design and implementation of social and cultural interventions.

Hekler et al [38] proposed models and theories for DBCI based on international experts' discussions (including behavioral, computer, and health scientists and engineers) and provided suggestions for developing models and theories that can be learned from DBCI and can provide references. The proposed framework provides state-space representations to define when, where, for whom, and for the person in which the intervention will have a targeted effect. *State* refers to an individual's state based on various variables, which define the *space* in which an action mechanism may affect. The state-space representation can be used to help guide theorization and determine interdisciplinary methodological strategies to improve measurement, experimental design, and analysis so that DBCI can match the complexity of real-world behavior changes.

### RQ2: What Are the Different Approaches to Provide Digital Interventions for Healthy Lifestyle and What Are the Essential Methods?

**Figure 2:**
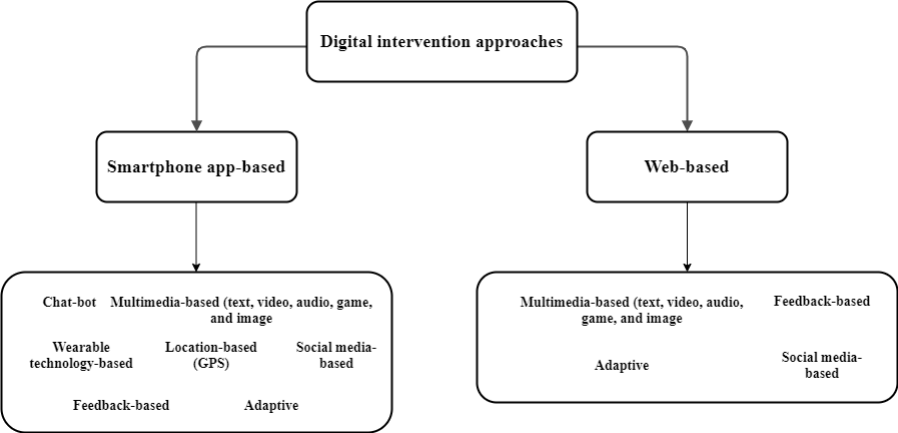
Structuring of digital intervention approaches for a healthy lifestyle.

In this systematic literature review, we identified the following 2 digital intervention approaches for a healthy lifestyle, and they are further structured in Figure 2: *smartphone app–based intervention (health monitoring and personalized recommendation generation)* [33,39-60,80-106] and *web-based intervention (web-based monitoring and self-management or self-reporting program)* [1,61-79,97-102].

In this systematic literature review, 35 studies targeted app-based interventions, 18 targeted web-based interventions, and 19 targeted both app-based and web-based interventions. The essential methods associated with both types of intervention approaches are listed in Textbox 4.

**Essential methods associated with digital interventions for healthy lifestyle.**

**Study and key methods associated with digital interventions**

- Lindwall et al [107]: motivation, satisfaction, self-determination, human-centered design, psychological needs (competence, autonomy, and relationship), and exercise-related behavior in selected motivational profiles
- Nicklas et al [108]: motivation and self-determination
- Yu et al [109]: self-monitoring for dietary intake—pen and paper documentation, self-check, and PDAs
- Karppinen et al [110]: motivation, persuasion with persuasive technologies, self-monitoring, goal setting, and evaluation
- Nouri et al [111]: user-centered design, observation, persuasive prompts, and feedback generation
- Sharpe et al [112]: efficacy evaluation, encouragement, and engagement
- O’Connor et al [113]: complexity analysis and identification of challenges
- Yardley et al [114]: feasibility study, usability study, beliefs, attitudes, needs, and conditions of people
- Fischer et al [105]: behavior change techniques, personal coaching, regular prompting, and efficacy evaluation
- Mitchell et al [53]: engagement, goal setting, incentives, and goal evaluation

### RQ3: What Is the Importance of a Digital Intervention to Promote a Healthy Lifestyle Targeting Obesity and Overweight?

In recent years, an increasing number of digital intervention approaches have been implemented to promote a healthy lifestyle in different age groups. This RQ found modest evidence for effective digital interventions to improve PA, diet, and habits to prevent obesity and overweight. In individuals who are overweight and obese, therapeutic weight control approaches contribute to clinically significant weight losses; however, due to limited access, expense, and time constraints, many people cannot engage in these face-to-face treatments. The advancement of several digital weight loss services has resulted in technological advances, such as universal access to the internet, expanded use of smartphones, and newer behavioral self-monitoring tools.

Verjans-Janssen et al [99] recognized the importance of implementing a long-term, locally relevant, holistic approach to promoting healthy weight status, stimulating the PA levels of children, and preventing them from wasting unnecessary time throughout school days on sedentary behaviors.

Brigden et al [115] designed interactive DBI for younger children based on the following characteristics: participation of parents, gaming functionality, additional therapist assistance, behavioral (rather than cognitive) approaches, and unique feedback and monitoring, shaping knowledge, repetition and substitution, and reward.

Nicklas et al [108] conceptualized a multi-exposure theory-based motivational theater, which can be an efficient behavior technique to improve preschool children's intake of vegetable dishes that can be conveniently disseminated to a large sample.

Lubans et al [36] used the Supportive, Active, Autonomous, Fair, and Enjoyable concepts to develop realistic strategies to engage young people with PA sessions to maximize involvement in PA and facilitate physical literacy by optimizing the results of affective, emotional, motivational, and movement skills.

Burrows et al [79] supported the need for web-based delivery of a balanced lifestyle program that addresses higher nutritional parental issues rather than infant weight. Parents were interested in a web-based family healthy lifestyle program and shared a desire for the program’s website to be easy to navigate and user-friendly, casual, but with personalized guidance and goal-setting opportunities.

Carrà et al [41] investigated *the Interactive Alcohol Risk Alertness Notifying Network for Adolescents and Young Adults (D-ARIANNA)*, a publicly accessible evidence-based eHealth app to estimate the current health risks by queries and fit-defined risk factors and include an overall risk score in percentage terms, accompanied by relevant images showing the main contributing factors in overview graphs and achievement.

Helle et al [57] conducted a study and identified 6 main behavioral risk factors as strong determinants of chronic diseases in adolescents (risky alcohol consumption, smoking, low diet, physical inactivity, sedentary behavior, and unhealthy sleep patterns). The study revealed that web and mobile technology interventions benefit adolescent participation, scope, and scalability to prevent the identification of health risk behaviors.

Stockwell et al [67] reported that PA and sedentary behavior are modifiable risk factors for lifestyle diseases and healthy aging; however, most of the older adults remain inadequately active. DBCIs can reach many older adults to promote PA and reduce sitting time. DBCIs may increase PA and physical function and reduce sedentary lifestyle and systolic blood pressure in older adults, but more high-quality testing is required.

According to Stephenson et al [40], machines, smartphones, and wearable technology resources can reduce the average sedentary time (minutes/day).

Weegen et al [62] showed that behavioral approaches were not successful without digital resources, and the integration of behavioral interventions with digital media proved to be an efficient way to stimulate PA.

Geidl et al [98] performed a recommendation generation study in adults with lifestyle diseases for PA and PA promotion over a week with a guideline of performing at least 150 minutes of aerobic PA with moderate intensity, 75 minutes of aerobic PA with vigorous intensity, or a combination of both. The PA and PA promotion guidelines advise adults impacted by lifestyle diseases and health providers on how much PA for adults with lifestyle diseases would be ideal. The guidelines provided the best strategies and approaches for growing low PA levels in adults with lifestyle diseases to professionals entrusted with PA promotion.

Gans et al [81] performed a study on 2525 worksite employees, and after 4 months, dietary fat intake decreased significantly with a multimedia-based (video) intervention strategy. Individually tailored videos helped office workers minimize dietary fat and increase fruit and vegetable consumption. Recently, to minimize sedentary behavior, technology-enhanced solutions such as mobile apps, activity monitors, prompting apps, SMS text messages, emails, and websites have been exploited.

Step-count sensors can improve walking, helping to tackle physical inactivity (pedometers, body-worn trackers, and smartphone apps). Chaudhry et al [87] assessed the influence of step-count monitors on PA in community-dwelling adults in randomized controlled trials, including longer-term results and discrepancies between step-count monitors and components of the intervention.

Muench et al [33] performed a multimedia-based (text) qualitative intervention to show the positive impact of digital triggers (such as SMS text messages, emails, and push alerts) in adults to change in curative conduct in health interventions. New technology apps for mobile health (mHealth) are emerging and provide the basis for fundamentally changing medical research, treatment practices, and scope.

Lin et al [43] conducted a web-based study on 4144 adults, collaborating with Quit Genius, an mHealth app focused on cognitive behavioral therapy that helps users quit smoking, to explore the successful nature of an mHealth digital app, which provides its users with substantial benefits and helps them modify their habits for a healthy lifestyle. The app's ability to improve users' hedonic well-being and inspire them mentally in their everyday lives was described as essential to help users quit smoking. The findings found that users whose well-being was improved via the app were 1.72 times more likely to quit smoking successfully.

Korinek et al [101] revealed that an adaptive phase target plus reward intervention using a mobile app appeared to be a feasible solution to increasing walking activity in overweight adults. Satisfaction with the app was strong, and the participants enjoyed having variable targets every day.

Mummah et al [32] tested the effect of a mobile app to increase vegetable consumption among overweight adults seeking to sustain weight loss. The findings showed the effectiveness of a mobile app in increasing the consumption of vegetables among overweight adults.

Hanze University [104] launched a health promotion initiative to enable workers to lead a less sedentary life. The use of an activity tracker for tracking the regular step count of participants was one of the program's measures. For a fortnightly coaching session, the regular move count acted as feedback. They argued that the use of machine learning in the process of automated personalized coaching might become an invaluable advantage. Individualized algorithms allow PA to be predicted during the day and provide the ability to intervene in time. Machine learning techniques empower automatic coaching and personalization.

Since attending a weight control program, many individuals who are overweight find it difficult to sustain weight loss. Self-weighing and telephone support are useful tools for weight loss monitoring. Partridge et al [82,84] and Sidhu et al [83] tested the efficacy of a weight maintenance program based on SMS text messaging to facilitate daily self-weighing in adults and found it to be effective for young men and women.

Ball et al [69] organized an incentive-based, promising web-based intervention study to increase PA and reduce sitting among adults (ACHIEVE: Active Choices IncEntiVE). They explored the effectiveness, appeal, and impact of offering nonfinancial incentives for inactive middle-aged adults to encourage increased PA, decreased sedentary time, decreased BMI, and blood pressure.

Franssen et al [90] performed a study on consumer wearable activity trackers to promote PA levels.

Oosterveen et al [72] conducted a qualitative analysis of eHealth behavioral interventions aimed at analyzing smoking rates, nutritional habits, alcohol consumption, PA levels, and obesity in young adults and revealed that because of their high level of use of technology, eHealth interventions have potential among young adults.

Therefore, this RQ reveals that digital interventions have the potential to promote a healthy lifestyle (regular PA, healthy habits, and proper dietary intake) in all age groups, for personal weight management. In therapeutic approaches, self-monitoring is a crucial part of digital intervention [73]. According to a study [73], participants participating in behavioral interventions have lost 8%-10% of their initial body weight. These results are considered positive based on studies suggesting that losses of 5% can yield positive health improvements such as reductions in triglycerides, blood glucose, blood pressure, improved blood lipid levels, and a reduction in the risk of developing type 2 diabetes for a person. It is difficult to sustain long-term weight reductions achieved through behavioral therapy, and a different set of skills may be needed for success following interventions [1,32,42]. Many teenagers have low diet and PA patterns, which in later life can contribute to the development of lifestyle diseases. Web-based networks provide an affordable means of providing health interventions, but their efficacy is poorly understood. Investigation of the locations of PA and dietary patterns can promote *setting-specific* lifestyle interventions and increase knowledge of contextual vulnerabilities to poor health. As future directions for digital weight management, distribution, and policy implications should be emphasized [131-133].

## Discussion

### Research Questions

*RQ1* helps to identify that behavioral theory, design thinking, evaluation, and the identification of limitations in the existing digital intervention frameworks are essential for successful, healthy lifestyle management. In contrast, *RQ2* reveals different approaches and methods associated with digital health interventions. Digital interventions for healthy lifestyle management have been categorized as discrete usefulness of advanced innovation applied to accomplish well-being goals and is executed inside digital well-being programs and information and communication technology frameworks, including communication channels, such as instant messages (SMS text message), alerts, and app-based notifications [116-118]. Digital intervention can motivate and stimulate individuals with self-tracking, goal setting, evaluation, and feedback or recommendation generation to promote a healthy lifestyle [118,119]. Different methods appear to impact health outcomes and usability. It would be interesting to test variants of component design and their impact on health outcomes and usability. Use of personalization to account for differences in preferences between groups of participants and even within groups of participants is essential in addition to cocreation between intervention developers and the target group [118-121]. Participants who attempted to self-manage their healthy lifestyle found that the most challenging part was to remain motivated [39]. They require apps that give them power and inspiration [39]. The study has confirmed that motivation is a multidimensional construct and people have different, sometimes competing, reasons for engaging in activities [39]. Moreover, human-centered analysis in digital intervention to study the intrinsic interactions of motivation and different regulations must be addressed. Despite the widespread use of mobile phones, digital literacy barriers are common among vulnerable people [39]. Participants have different participation levels in various activities, from higher to lower levels of participation. Researchers using traditional user-centered design methods should routinely measure these communication domains in their end-user samples. Future research should replicate these findings to a larger sample through direct observation, and persuasive prompts may be more effective in providing feedback to those with communication difficulties. *RQ3* summarizes the evidence on the importance of digital intervention that were exclusively directed at promoting healthy lifestyles, especially in children, adolescents, adults, and older adults. In the next section, we discuss the importance of digital intervention on healthy lifestyle promotion elaborately.

### Digital Intervention and Healthy Lifestyle

A healthy lifestyle is a lifestyle that reduces the risk of severe illness or early death. Not all diseases are preventable, but a large proportion of deaths can be avoided, especially lifestyle or noncommunicable diseases. According to the *Harvard Medical School*, the key lifestyle factors to be monitored are healthy diet, healthy PA level, healthy body weight, no tobacco consumption, and moderate alcohol intake [31]. According to *RQ3*, digital intervention can have a significant impact on healthy lifestyle management.

A study conducted by Steene et al [86] found significant country- and region-specific variations in PA and sedentary time in the European population, with lower PA levels. Boys in all age groups were more aggressive and less sedentary. At about 6 to 7 years of age, the initiation of age-related decline or leveling-off of PA and rise in sedentary time begins to become evident [86]. In children and adolescents, sedentary behavior strategies successfully decrease screen time; however, the scale of the effect tends to be limited [129]. The potential of digital intervention in older age groups outside of occupational settings and during sedentary leisure time must be examined in future studies. The sustainability of lifestyle changes in a positive direction remains a challenge [129]. Mobile apps to improve PA in young adults should include customized and personalized feedback and provide a coaching feature [58-60]. It is essential to create a well-oriented and easy-to-use interface with the ability to customize the app. The new area of mHealth is mHealth apps that target willing participants to enhance self-management of chronic conditions [58-60]. However, we found that only a small fraction of the mHealth apps available had been reviewed, and the amount of evidence was of inferior quality [58-60]. Improving the quality of evidence includes supporting prerelease app performance monitoring, designing few experiments, and performing better reviews with a rigorous risk of bias assessments [43,115]. Without enough evidence to back it up, for some time to come, digital intervention and app practicability will stall in their infancy [43,115,121]. Evidence suggests that an unhealthy lifestyle is associated with poor health outcomes [40,63]. It can have severe implications for health and well-being at any age, [63,64,126]. Therefore, there is a need to review the effects of multicomponent, complex interventions that include effective unhealthy lifestyle reduction strategies. We must focus on optimizing the effects of an intervention. Future intervention studies should use more rigorous methods to improve the quality of studies, considering larger sample sizes, randomized controlled designs, and valid and reliable lifestyle measurements. An overview of intervention development methods can help researchers understand various existing methods and comprehend the range of actions taken in intervention development before evaluating feasibility or pilot interventions [32,126,135].

One way to encourage PA and enhance health is to change the physical environment, but research on intervention efficacy is mixed [92-94]. Theoretical perspectives and conceptual problems are used in evaluative studies, and related literature can contribute to these inconsistencies [92-94]. Environmental and policy initiatives are socially incorporated into the framework and function through it. Therefore, a philosophical viewpoint must be considered and should be understood by evaluators. Future research should aim to explain how interventions function across disciplinary fields by considering these structures, the context in which interventions occur, and the measurable and unmeasurable mechanisms that might work [92-94,98]. It can be beneficial to promote health-based actions, such as PA, by using innovative and interactive media-based health education [92-94,98,134]. Therefore, to successfully influence behavior, it is essential to establish user-based techniques and reinforce the theories and hypotheses of behavioral change based on digital media. Step-count tracking [87] leads to improvements in short- and long-term step counts. There is no proof that either wearable sensors or smartphone apps, or extra counseling or incentives have additional benefits over more straightforward approaches focused on the pedometer [63,64]. To overcome the public health issue associated with physical inactivity, basic step-count tracking strategies should be prioritized. In general, it is not clear how self-reported sedentary behavior (eg, questionnaires, logs, and momentary ecological evaluations) compares with system measurement measures (eg, accelerometers and inclinometers) [63,64]. Evidence from this study indicates that when compared with system tests, single-item self-report measures typically underestimate sedentary time [63,64]. Therefore, to evaluate the reliability and validity of different self-report measures for evaluating sedentary activity, studies should exercise caution when comparing associations between various self-report and system measures with health performance.

In addition, video and adapting technologies have been effective in diet change measures; however, these methods have never been combined with researching personalized video efficacy [81]. Theory-based mobile interventions could provide a low-cost, scalable, and efficient approach to improving dietary habits and preventing associated chronic diseases. To encourage a healthy dietary pattern, nutrition messages or nutrient labeling, offering healthier choices, and portion size management of unhealthy foods have been potentially effective strategies in tertiary education environments [75]. The reduction in rates and the increased availability of nutritious choices in conjunction with nutrition knowledge have contributed to changes in dietary habits [75]. Further studies comparing the long-term efficacy of the climate and the combination of environmental policies to improve health outcomes are warranted. Dietary consumption has increased by increasing the availability of nutritious foods and reducing the portion size of unhealthy foods. In terms of modifying overall dietary patterns, the existing evidence base is misleading, as rising intake of desirable food groups was more effective than reducing unfavorable food habits, and fruit or vegetable intake and sugar-sweetened beverage consumption are the most notable observed changes [75,79]. Social support, followed by a demonstration of conduct, self-monitoring, goal setting, and feedback, is the most popular digital health behavior intervention [55,67,85,96]. In addition, a customized Facebook-based obesity prevention program for teenagers in Korea (Healthy Teens) [76] revealed usability problems in terms of material, appearance, and navigation. Facebook [76,96] has tremendous potential in promoting communication and engagement with immigrant teens, considering its prominence among adolescents. Interventions focused on social media (eg, Facebook) [66,96] are productive in facilitating meaningful improvements in adolescent eating habits. However, more research is required to explore effectiveness variations based on component tailoring, best use stimuli to promote behavior change over time, and keep people involved in changing physical health behavior. The first step is to dismantle digital triggers into their parts and reassemble them according to their goals for improvement.

PA, sedentary time, and dietary habits vary across homes, schools, and other locations [95]. Health habits vary depending on the place or environment in which the participants are [95]. Although eating habits are typically more beneficial in home or school locations, PA is usually low and sedentary time in these locations is higher [95]. To optimize health habits in each area, digital interventions that address the various locations in which participants spend time and use location-specific behavior change techniques should be explored [95]. Among young people, binge drinking is prevalent [41]. eHealth technologies [41] are appealing to them and can be useful in raising awareness. However, to make eHealth apps suitable for longer-term effects, additional components, including daily feedback and repeated administration by different multimedia interventions, may be needed. Mass media campaigns [65] for smoking or tobacco programs are also effective over long periods. Digital interventions have been associated with decreased drinking and smoking frequency, with a slight yet persistent impact on teenagers and adults. Protective effects against alcohol and tobacco [65] use can be demonstrated through digital initiatives focused on a combination of social maturity and approaches to social influence. Evidence tends to be mixed with internet-based interventions, policy proposals, and incentives, and requires further study. Various distribution systems can enhance the effects of alcohol or tobacco misuse among teenagers and adults, including interactive platforms and policy initiatives. Adolescents are easily accessible by digital media and can represent a scalable and inexpensive opportunity to engage this audience in changing behavior [65]. Smartphone-based interventions [39-59] (such as apps, SMS text messages, sports, multicomponent interventions, emails, and social media) are readily available, inexpensive, and use tools already used by most teenagers. Therefore, it is essential to perform and publish high-quality academic literature studies and formally evaluate apps that have already been developed to inform the creation of potential interventions to change behavior. Essential improvements in behavior were also seen when interventions involved schooling, setting goals, self-monitoring, and parents' participation. Digital approaches [66] that include education, goal setting, self-monitoring, and parental participation can affect adolescents' meaningful health behavior changes. Most of the evidence relates to goal setting, further research into alternative media is needed, and it is essential to assess longer-term effects. There is a lack of evidence on the cost-effectiveness of digital health initiatives, and these data should be recorded in future trials. The young population has broadly embraced social media, so health researchers are searching for ways to exploit this social media involvement to deliver programs and health promotion campaigns [66,96]. In young adults, weight gain and suboptimal dietary choices are popular, and social media can be a possible instrument for encouraging and supporting healthy choices. The dissemination of information is now an appropriate use of social media by young adults. Careful evaluation is needed to use social media effectively for social support, either by private or by public sites, as its efficacy has yet to be demonstrated in experimental designs. In digital intervention studies aimed at manipulating weight, concerns about public social media use can lead to low engagement with social media [66,96,115]. Future research should explore how to use social media to better connect with young adults, how to use social media more efficiently to help young adults, and how to encourage social and peer-to-peer support to make healthy choices.

The systematic literature review has revealed that the identified digital intervention methods affect lifestyle behavior outcomes, focusing on PA, diet, habit, and associated primary and secondary health outcomes, such as fitness, motivation, reduced sedentary bouts, weight augmentation or weight status, blood pressure, glycemic responses, lipid profile, and quality of life in different study groups, as explained in the next section. Effectiveness (n=55), conceptualization (n=24), selection of appropriate methodology (n=25), effective and technological engagement (n=24), selection of strategies or techniques (n=20), motivation (n=15), feedback generation (n=15), environment (n=10), satisfaction (n=6), credibility (n=6), digital literacy (n=6), self-determination (n=5), and user-centric design (n=4) were responsible for the successful implementation of digital interventions for healthy lifestyle management. Here, *n* signifies the total number of overlapped studies in which the respective parameters are identified. Digital interventions [84,106,124] focused on mobile phone apps may be an acceptable and efficient way of encouraging weight loss in people who are overweight or obese. Digital health coaching can be a revolutionary approach to reduce barriers to access to much-needed weight loss therapies for obesity, given the ubiquity of mobile phones.

### Strength and Limitation

A systematic literature review revealed that a healthy diet, healthy habits, and regular PA are powerful tools for reducing obesity and associated health risks. These findings bolster the use of digital interventions as a preventive option for obesity and overweight. Therefore, behavior change should be given the highest preference to avoid severe health damage. Planned digital interventions may potentially change growing negative behavior in humans with the adoption of persuasion, observation, goal evaluation, evidence-based personalized recommendation generation, health risk predictions (decision-making), automation, motivation, pragmatism, and trust. Developing and maintaining an empathetic relationship is perhaps the most critical determinant of successful digital intervention [106,124]. It is essential to know the participant first, and the interaction aspects are challenging owing to the delay in reaction time (both ways). Health care professionals need to ensure both relationship communication and goal-oriented coaching when using such digital intervention solutions. In the future, the quality of the interaction between the system and the participant will require attention if participants are to fully benefit from collaboration in digital intervention programs.

Digital intervention for healthy lifestyle management has great potential as a scalable tool that can improve health and health care delivery by improving effectiveness, efficiency, accessibility, security, and personalization. Therefore, a knowledge base must be accumulated to provide information for developing and deploying digital health interventions [116,118]. However, the evaluation of digital health interventions poses unique challenges. Methodological limitations, selection of appropriate intervention methods, evaluation of efficacy, limitations of research on different populations, loss to follow-up, attrition rate, lack of participation in tracking, financial incentives and intervention burdens (long term or short term), digital literacy, technical participation, personalization, useful evidence-based automatic tailored lifestyle recommendation generation (intervention design), research heterogeneity, meta-analysis, cost-effectiveness (technical and financial feasibility analysis), trial selection, trial recruitment, scalability, accessibility, ethics, policy development, cyberbullying, safety, trust, user-centeredness, adoption of health care and collaboration methods that promote cooperation, unsustainable growth in complexity, and efficacy evaluation are some of the existing limitations of digital health interventions that should be overcome in existing research [126,128]. Although new technologies and rapidly changing technologies pose many unsolved problems, the broad consensus is that successful intervention design requires user-centered iterative development methods, hybrid methods, and in-depth qualitative research to gradually improve interventions to satisfy users. Therefore, conceptual participation (effective engagement) is essential to understand the relationship between the involvement in digital interventions and required behavioral changes and to achieve population-level benefits. Interventions must be delivered effectively at scale. Small effect sizes and high dropout rates [78,126] often affect web-based computer-tailored interventions, particularly among people with a low education level. The results and attractiveness of these remedies can theoretically be enhanced by using videos as a delivery format. The most successful and most appreciated intervention was the web-based video version of the computer-tailored obesity prevention intervention. Future research needs to analyze whether the results are sustained in the long run and how to maximize the intervention.

### Conclusions

Digital intervention in health care is the intersection of health care, behavior science, computing, and engineering research and requires methods borrowed from all these disciplines. Digital interventions have effectively improved many health conditions and health behaviors; besides, they are increasingly being used in different health care fields, including self-management of long-term conditions, prevention of lifestyle diseases, and health promotion. In low-resource primary care environments, digital health strategies can be useful for preventing obesity. To minimize obesity and chronic disease risk among medically vulnerable adults in the primary care environment, digital health intervention uses an advanced digital health approach. The lack of user involvement hinders the full potential of digital interventions. There is an urgent need to develop effective strategies to promote user participation in digital interventions. One potential method is to use technology-based reminders or personalized recommendation generation. Compared with no strategy, technology-based strategies can promote participation. However, the findings of this systematic literature review should be understood with prudence, as only a few qualified studies have been identified for review, and the results are heterogeneous. The number and dates of studies indicate that a digital health intervention strategy is an emerging field. More research is needed to understand what strategic features are useful, their cost-effectiveness, and their applicability to different age groups. The results of this literature review will help to understand the concepts and parameters behind different DBI methods, thereby developing, testing, and evaluating the performance of a useful digital intervention in the future.

## Abbreviations

DBCI: digital behavior change intervention
DBI: digital behavior intervention
mHealth: mobile health
PA: physical activity
RQ: research question
SANRA: Scale for the Assessment of Narrative Review Articles
TUDER: Targeting, Understanding, Designing, Evaluating, and Refining
